# Using patient-collected clinical samples and sera to detect and quantify the severe acute respiratory syndrome coronavirus (SARS-CoV)

**DOI:** 10.1186/1743-422X-4-32

**Published:** 2007-03-27

**Authors:** Zhongping He, Hui Zhuang, Chunhui Zhao, Qingming Dong, Guoai Peng, Dominic E Dwyer

**Affiliations:** 1 Beijing Ditan Hospital, Beijing 100011, People's Republic of China; 2 Capital University of Medical Sciences Affiliated Beijing YouAn Hospital, Beijing 100054, People's Republic of China; 3 Department of Microbiology, Peking University Health Science Center, Beijing 100083, People's Republic of China; 4 Centre for Infectious Diseases and Microbiology Laboratory Services, Institute of Clinical Pathology and Medical Research, Westmead Hospital, Westmead NSW 2145, Australia

## Abstract

**Background:**

Severe acute respiratory syndrome (SARS) caused a large outbreak of pneumonia in Beijing, China, in 2003. Reverse transcriptase polymerase chain reaction (RT-PCR) was used to detect and quantify SARS-CoV in 934 sera and self-collected throat washes and fecal samples from 271 patients with laboratory-confirmed SARS managed at a single institution.

**Results:**

SARS-CoV detection rates in sera were highest in the first 9 days of illness, whereas detection was highest in throat washes 5–14 days after onset of symptoms. The highest SARS-CoV RT-PCR rates (70.4–86.3%) and viral loads (log_10 _4.5–6.1) were seen in fecal samples collected 2–4 weeks after the onset of clinical illness. Fecal samples were frequently SARS-CoV RT-PCR positive beyond 40 days, and occasional sera still had SARS-CoV detected after 3 weeks of illness.

**Conclusion:**

In the context of an extensive outbreak with major pressure on hospital resources, patient self-collected samples are an alternative to nasopharyngeal aspirates for laboratory confirmation of SARS-CoV infection.

## Background

Severe acute respiratory syndrome (SARS) emerged in late 2002, with more than 8096 cases reported by April 21 2004 by the World Health Organization, mostly in China (5327), Taiwan, Hong Kong SAR, Singapore and Canada. There were 774 deaths and a mortality of 9.6% [1]. The largest outbreak was in Beijing, with over 2,521 cases [2]. The SARS-associated coronavirus (SARS-CoV) was identified as the causal agent following its isolation and detection by electron microscopy and reverse transcriptase polymerase chain reaction (RT-PCR) from a range of clinical specimens. Serological evidence of infection has been found in most patients fitting the clinical definition of SARS [3-6]. The clinical, radiological, and laboratory findings of SARS from Beijing and elsewhere have been described previously [2,5,7-12].

The aim of this study was to detect and quantify SARS-CoV using RT-PCR in sera and throat washes and stools self-collected by 271 patients with laboratory confirmed SARS managed at a single institution. These samples were collected during the extreme pressure of the Beijing SARS outbreak in the context of healthcare worker concern about the safety of collecting nsaopharyngeal aspirates (NPAs) from ill patients.

## Results

Between March 26-May 31 2003, 304 patients fitting the case definition of probable SARS were hospitalized. Of these, 271 were laboratory confirmed following the detection of SARS-CoV-specific IgM and/or IgG antibody by immunofluorescence [6] and/or by the detection of SARS-CoV RNA by RT-PCR.

The mean age of the cohort was 36 ± 16 years. There were 92 (33.9%) healthcare workers who acquired SARS, including 51 nurses, 30 physicians, 5 logistics staff, 3 pharmacists and 2 laboratory technicians (one of whom was believed to be infected after handling sputum and stool samples from SARS patients in a diagnostic laboratory). A total of 112 people were infected following exposure to SARS patients in the hospital setting, either as healthcare workers, patients or visitors, and another 62 cases were household contacts of known SARS cases. Common clinical features on admission included fever (100%), subjective shortness of breath (57%), nonproductive cough (55%), malaise (52%), myalgia (38%), headache (30%), dyspnea (21%), chills (17%), diarrhea (11%) and sore throat (6%). The mortality rate was 9.2% (25/271) amongst laboratory-confirmed cases.

Sera, throat washes and stool samples were tested for SARS-CoV RNA by RT-PCR. A total of 614 sera (ranging from 1–7 per patient) were collected 1–78 days after the onset of illness from 271 cases. Overall, 31.3% (192/614) of sera had detectable amounts of SARS-CoV RNA detected, with viral loads ranging from 10^1^-10^3 ^copies/ml serum (Table 1). Sera collected within 9 days of disease onset were more likely to be RT-PCR positive (54%) than later in the disease course, although SARS-CoV RNA was still occasionally detected in sera out to 24 days of illness.

**Table 1:** Detection and quantitation of SARS-CoV RNA by RT-PCR in sera.

Days after onset	Samples	Positive (number, %)	Viral load (log_10 _copies/ml ± SD)
1–4	76	38 (50)	2.74 ± 0.88
5–9	154	87 (56.5)	2.78 ± 1.04
10–14	129	39 (30.2)	2.58 ± 1.02
15–19	88	24 (27.3)	2.27 ± 0.85
20–24	24	4 (16.7)	2.11 ± 1.04
≥25	143	0	-
Total	614	192 (31.3)	2.65 ± 0.99

A single throat wash was self-collected by 96 patients 1 to 35 days after the onset of disease. A total of 50 (52.1%) had SARS-CoV RNA detected by RT-PCR (Table 2), with viral loads ranging from 10^1^-10^5 ^copies/ml wash fluid. The highest detection rate was 61% in throat washes collected between days 5 and 14.

**Table 2:** Detection and quantitation of SARS-CoV RNA by RT-PCR in throat washes.

Days after onset	Samples	Positive (number, %)	Viral load (log_10 _copies/ml ± SD)
1–4	8	3 (37.5)	4.73 ± 0.45
5–9	45	27 (60)	3.59 ± 1.36
10–14	24	15 (62.5)	3.33 ± 1.37
15–19	11	5 (45.5)	1.88 ± 1.00
20–24	4	0	-
≥25	4	0	-
Total	96	50 (52.1)	3.45 ± 1.39

Of 224 stool samples self-collected by 188 patients (1–2 samples each), 127 (56.7%) had SARS-CoV RNA detected by RT-PCR (Table 3). Stool samples were not collected in the first 10 days of illness, but high rates of SARS-CoV RNA detection (44/51, 86.3%) were seen in stools collected between 10 and 19 days after onset. Viral loads in stool were as high as 10^10 ^copies/g feces from day 10. Fecal samples collected 40 days or more after onset of disease contained SARS-CoV RNA in 29.8% (17/57) of samples, with a mean load of 7000 copies/g feces. The fecal load of SARS-CoV was at least between 2 and 3 logs higher than in throat washes or sera at comparable time points.

**Table 3:** Detection and quantitation of SARS-CoV RNA by RT-PCR in fecal samples.

Days after onset	Samples	Positive (number, %)	Viral load (log_10 _copies/g ± SD)
10–19	51	44 (86.3)	6.06 ± 2.05
20–29	54	38 (70.4)	4.51 ± 1.23
30–39	62	28 (45.2)	3.82 ± 1.44
40–53	57	17 (29.8)	3.57 ± 1.25
Total	224	127 (56.7)	4.37 ± 1.61

## Discussion

The clinical features of this cohort of 271 patients managed at a single institution were similar to those reported elsewhere [2,5,7-12], although diarrhea was present in only 11% of patients compared to rates of 20–73% reported in studies from Hong Kong and Canada [7-9]. Like other SARS outbreaks, many cases (41.3%) were acquired after exposure in the hospital environment, with healthcare workers providing 34% of cases at this institution. Of note was a case of SARS possibly acquired in a diagnostic laboratory. There have since been a number of cases acquired in research laboratories [1].

Detection of SARS-CoV RNA by RT-PCR is only moderately useful in the early diagnosis of SARS, as the maximal viral load and RT-PCR sensitivity occurs in the second week of illness [9]. In addition, the sensitivity of SARS-CoV RT-PCR on specimens collected from different sites and at different time points in the illness varies. Testing more than one clinical specimen increases the likelihood of obtaining a positive RT-PCR result. In one large study, 60% of patients with clinical SARS had a positive SARS-CoV RT-PCR in one or more clinical specimens, with the highest detection rates in sputum (55.6%), NPAs (29.6%) and nose/throat swabs (20%) collected within the first 5 days of illness [10]. We found that the likelihood of a positive SARS-CoV RT-PCR was similar in serum (54.3%) and throat washes (56.6%) in the first 9 days of illness. We found the peak of SARS-CoV detection in throat washes to be between days 5 to 14, where 60.8% (42/69) of samples were positive, similar to reported rates in other respiratory specimens [9,10]. The viral loads in throat washes decreased over time and were at levels between those in feces and sera at similar time points.

In other studies, throat swabs were RT-PCR positive in 37.5% of probable SARS cases, reaching 50–60% on days 7–10, and consistent with earlier studies showing peaks of virus shedding in the respiratory tract in the second week of illness [9,11]. High viral loads were seen in NPAs in 14 patients with SARS, mainly in the second week of illness [9]. The use of patient self-collected throat washings may reduce risks to healthcare workers, although lower respiratory tract samples such as sputum, NPAs or bronchoalveolar lavage fluid are likely to have higher viral loads and offer increased likelihood of SARS-CoV detection by RT-PCR. We were unable to correlate viral loads in the various clinical samples with ability to isolate virus or transmission to other people; whether viral load in the respiratory tract correlates with 'super-shedding' events is uncertain.

Although overall SARS-CoV detection rates and viral load in throat washes and stools were higher than in the serum, serum SARS-CoV RT-PCR is a useful investigation early in the illness as we found that 50% of sera had SARS-CoV detected in the first four days of illness. One study of sera from 8 probable SARS patients found a detectable SARS-CoV load ranging from 2 × 10^3 ^to 1 × 10^4 ^copies/ml serum in 50% of the samples, but not after 12 days after onset [13]. Of interest was that occasional serum samples from individuals remained SARS-CoV RT-PCR positive (with moderate viral loads) over three weeks after onset of illness, a feature noted in another study [14].

High rates of SARS-CoV RT-PCR detection (as high as 86.3% between days 10–19) and high viral loads were found in fecal samples in the second to fourth weeks of disease. Rates of SARS-CoV detection in fecal samples began to decrease after one month, although many stools were still SARS-CoV RNA positive 40 days or more after the onset of the clinical illness. The SARS-CoV load in fecal samples collected after 40 days were higher than the peak load seen in sera collected early in disease, and comparable to the viral load in throat washes in the second week of illness. Both lower (27% in fecal samples collected 11–20 days after onset) and similar high detection rates (over 80% in stools collected 11–16 days after onset) have been reported elsewhere, as have fecal samples positive 40 days or more after onset [10,14]. Despite the high SARS-CoV load in feces, diarrhea was not a prominent clinical feature in this cohort. Long-term fecal viral shedding may be an additional source of community spread of SARS, although the infectivity of feces may be better assessed with virus isolation.

Direct comparisons of the sensitivity and specificity of RT-PCR for the detection of SARS-CoV are hampered by the use of different types of clinical specimens, RNA extraction procedures and different RT-PCR techniques. The first published interlaboratory comparison showed sensitivities of 61% and 68% for 72 NPAs, 65% and 72% for 54 throat swabs, 50% and 54% for 78 urine samples and 58% and 63% for 19 stool specimens, with an overall specificity of 100% [15]. To date, no significant differences in the sensitivity and specificity of various commercial and in-house RT-PCR or other molecular assays have been reported [16-18].

## Conclusion

SARS-CoV infection results in a severe respiratory disease. It causes significant nosocomial infection and requires aggressive infection control practices rarely used for other causes of atypical pneumonia. Laboratory confirmation of SARS is crucial in the management of patients presenting with pneumonia, particularly as the clinical features of SARS make it difficult to distinguish from other causes of atypical pneumonia. Molecular methods for SARS diagnosis are useful, although their value is affected by the observation that maximal viral shedding occurs after the first week of illness rather than at the initial clinical presentation. The SARS outbreak was characterized by high infection rates in healthcare workers; patient self-collected specimens such as throat washes or feces, or serum may pose less risk to healthcare workers, particularly in the context of concerns about nosocomial acquisition. Although NPAs and other lower respiratory tract samples are the sample of choice for suspected respiratory viral infections, patient self-collected specimens are suitable for RT-PCR. Thus they offer diagnostic value, especially in SARS where the peak of viral shedding is after the first week of illness, and this sampling approach may reduce the safety issues of healthcare workers collecting NPAs. Patient self-collected specimens may be less appropriate for common seasonal respiratory virus infections such as influenza, where viral shedding is maximal at clinical presentation and virus is rarely detected outside the respiratory tract. Accurate and rapid laboratory diagnosis will become even more important as SARS becomes less common, or in the event of new outbreaks of SARS, especially if influenza or other seasonal respiratory viruses are co-circulating.

## Methods

This study was conducted during the first three months (March-May 2003) of the SARS outbreak in Beijing, China, where Ditan Hospital was designated as a 'SARS hospital', meaning that suspected SARS patients were transferred and managed at this institution. The study was approved by the Ethics Committee of the Beijing Ditan Hospital. The clinical case definition of probable SARS included a fever of ≥38°C, cough or shortness of breath, new pulmonary infiltrates on chest radiography, and close contact with a suspect or probable SARS case. Day 1 was defined as the day of fever onset.

Sera, throat washes and feces were collected from hospitalised patients for testing with a quantitative SARS-CoV RT-PCR. As the early phase of the outbreak in Beijing had involved many healthcare workers [2], patient self-collected throat washes and fecal samples were used to minimize further nosocomial transmission. For throat washes, patients were given 10 ml of sterile 0.9% NaCl, asked to gargle for 30 seconds then spit the fluid into a 20 ml sterile plastic screw-topped plastic container. Patients were also asked to collect approximately 1 cm^3 ^feces and place it into a 20 ml sterile screw-topped plastic container.

Five ml of the throat wash was centrifuged at 10,000 g for 10 minutes, then the supernatant further centrifuged at 20,000 g for 1 hour. Ten ml of 0.01 M phosphate buffered saline (pH 7.2) was used to dilute the fecal sample, then 5 ml was centrifuged at 10,000 g for 10 minutes. The supernatant was further centrifuged at 20,000 g for 1 hour. RNA was extracted from the remaining 100–300 μl of the throat wash and fecal pellets using Trizol (Invitrogen, Beijing, PR China). 700 μl of sera were centrifuged at 20,000 g for 1 hour, the supernatant removed and RNA extracted from the remaining 100–300 μl pellet using Trizol.

SARS-CoV RNA was detected in throat washes, stool and blood using a fluorescence quantitative RT-PCR assay (ShenZhen PJ Biotech Company, Shenzhen, Guangdong Province, PR China), according to the manufacturer's instructions and performed on a BioRad iCycler thermal cycler (Bio-Rad Laboratories, Beijing, PR China). The SARS-CoV pol region primers used were P1 sense 5'GTTCTTGCTCGCAAACATAACACTT3' (position 15279–15303 in SARS-CoV Urbani strain, Genbank accession number AY278741), P2 antisense 5'AACAGCTTGACAAATGTTAAAGACA3' (15446–15470) and probe 5'TGTGTGGCGGCTCACTATAT3' (15373–15392). Internal controls were used in all runs, and no evidence of PCR inhibition in clinical samples was detected. Testing for other respiratory viruses was not carried out in this cohort of patients as they fitted the SARS clinical case definition during the outbreak. The PCR assay was negative when performed on RNA or DNA extracted from influenza A and B, rhinovirus, respiratory syncytial virus and adenovirus isolates, and on plasma collected from otherwise healthy hepatitis C and B infected individuals (data not shown).

Manipulations were carried out in a BSL2 facility with BSL3 practices. SARS-CoV isolation was not attempted on clinical samples during the outbreak due to safety concerns and time constraints.

## Competing interests

The author(s) declare that they have no competing interests. The study was funded by the Beijing Ditan Hospital.

## Authors' contributions

ZH and DD reviewed the data and wrote the manuscript. ZH, QD, CZ, GP, and HZ collected the samples and clinical data, and undetook the performance of the molecular assays. DD was a short-term consultant with the World Health Organisation in Beijing, China during the SARS outbreak in 2003.
